# A Network Analysis of Perinatal Depression, Anxiety, and Temperaments in Women in the First, Second, and Third Trimesters of Pregnancy

**DOI:** 10.3390/jcm13133957

**Published:** 2024-07-05

**Authors:** Marianna Mazza, Caterina Brisi, Giorgio Veneziani, Francesco Maria Lisci, Ilenia Sessa, Marta Balocchi, Sara Rossi, Enrico Di Stasio, Giuseppe Marano, Francesca Abate, Maria Benedetta Anesini, Gianluca Boggio, Michele Ciliberto, Valeria De Masi, Cecilia Falsini, Ester Maria Marzo, Carla Avallone, Annamaria Serio, Angela Gonsalez del Castillo, Georgios Demetrios Kotzalidis, Daniela Pia Rosaria Chieffo, Antonio Lanzone, Giovanni Scambia, Carlo Lai, Gabriele Sani

**Affiliations:** 1 Unit of Psychiatry, Fondazione Policlinico Universitario Agostino Gemelli IRCCS, 00168 Rome, Italy; caterinabrisi@libero.it (C.B.); fmlisci@gmail.com (F.M.L.); sara.rossi1349@gmail.com (S.R.); giuseppemaranogm@gmail.com (G.M.); abatefranci@gmail.com (F.A.); mbenedetta@hotmail.it (M.B.A.); gianlu88us22@hotmail.it (G.B.); micheleciliberto@libero.it (M.C.); valeriademasi95@gmail.com (V.D.M.); ceciliafal@virgilio.it (C.F.); estermarzo7@gmail.com (E.M.M.); giorgio.kotzalidis@gmail.com (G.D.K.); gabriele.sani@unicatt.it (G.S.); 2 Department of Neurosciences, Università Cattolica del Sacro Cuore, 00168 Rome, Italy; 3 Department of Dynamic and Clinical Psychology, and Health Studies, Sapienza University of Rome, Via Degli Apuli 1, 00185 Rome, Italy; giorgio.veneziani@uniroma1.it (G.V.); carlo.lai@uniroma1.it (C.L.); 4 Unit of Clinical Psychology, Fondazione Policlinico Universitario Agostino Gemelli IRCCS, 00168 Rome, Italy; ileniasessa3@gmail.com (I.S.); marta.balocchi@hotmail.it (M.B.); annamaria.serio@guest.policlinicogemelli.it (A.S.); angela.gonsalezdelcastillo@guest.policlinicogemelli.it (A.G.d.C.); danielapiarosaria.chieffo@unicatt.it (D.P.R.C.); 5 Department of Basic Biotechnological Sciences, Intensive Care and Perioperative Clinics Research, Fondazione Policlinico Universitario Agostino Gemelli IRCCS, Università Cattolica del Sacro Cuore, 00168 Rome, Italy; enrico.distasio@unicatt.it; 6 Department of Neurosciences, Mental Health, and Sensory Organs (NESMOS), Sapienza University of Rome, 00189 Rome, Italy; 7 Department of Gynaecology and Obstetrics, Fondazione Policlinico Universitario Agostino Gemelli IRCCS, Università Cattolica del Sacro Cuore, 00168 Rome, Italy; antonio.lanzone@unicatt.it (A.L.); giovanni.scambia@policlinicogemelli.it (G.S.)

**Keywords:** pregnancy, perinatal depression, anxiety, temperament, network analysis

## Abstract

**Background/Objectives:** Although depression and anxiety are found to be affected by temperaments, little research has studied these relationships in pregnancy. The present study explored the associations among perinatal depression (PD), anxiety dimensions (state, trait, and generalized anxiety disorder (GAD)), and temperaments between women in the three trimesters of pregnancy through a network analysis approach. Moreover, differences in the severity of PD and anxiety between women in the three trimesters were evaluated. **Methods:** Women in first (N = 31), second (N = 184), and third (N = 54) trimesters of pregnancy were recruited in the present cross-sectional study. The network analysis included PD, anxiety dimensions, and temperaments. Three network models were estimated, and ANOVAs evaluated the differences in the severity of PD and anxiety, including trimesters as a between-subject factor. **Results:** PD and GAD were the nodes most strongly connected across the three groups. Cyclothymic, depressive, and anxious temperaments were most frequently associated with PD and GAD. Hyperthymic temperament was in the periphery of the three networks. Lastly, women in the first trimester had the highest severity of PD and GAD. **Conclusions:** PD and GAD showed the strongest associations. Anxiety dimensions had positive associations with PD and GAD, suggesting their role as possible risk factors. Temperaments were differently associated within the network between the three groups. Clinical interventions during pregnancy should target the central variables, considering their direct and indirect relationships.

## 1. Introduction

Pregnancy marks a significant transition to motherhood, characterized by considerable physical and psychological changes [1,2]. This period is particularly susceptible to the development of mental disorders [3]. Depression, defined as a mood disorder characterized by persistent sadness and loss of interest, is one of the most common disorders affecting pregnant women from conception to 12 months postpartum [4]. Indeed, although feelings of weepiness and labile emotions, the so-called “baby blues”, are common [5], previous epidemiological research has reported that 19.7% of pregnant women suffer from depression [6]. A previous review focused on longitudinal studies reported an average prevalence rate of 17% for perinatal depression, which was found to be a predictor of postpartum depression [7,8].

### 1.1. Background of Perinatal Depression and Anxiety

In this context, it was found that perinatal depression tends to show strong associations with anxiety [9,10,11]. In the postnatal period, significant comorbidity between postpartum depression and anxiety has also been shown, and this seems to be influenced by factors such as perceived support and being primiparas [12]. Furthermore, women experiencing postpartum depressive and anxiety symptoms in comorbidity reported a higher rate of health problems compared to those reporting depression or anxiety alone [13]. According to the literature, the prevalence of perinatal anxiety ranges from 13 to 21% [14]. Trait anxiety, a stable tendency to experience anxiety in various situations, has been identified as a significant risk factor for perinatal depression and predicts pregnancy-specific anxiety [15,16,17,18]. Despite these findings, research on prenatal anxiety showed inconsistent results, considering that anxiety could be manifested by moderate symptoms, such as tension, as well as anxiety disorders—such as generalized anxiety disorder (GAD)—where the outcomes examined significantly influence the results [19,20]. Moreover, it has been seen that the prevalence and intensity of psychiatric symptoms differ depending on the trimester of pregnancy [21,22,23]. In this regard, depression symptoms seemed to be more intense in the first trimester, and their intensity decreased in the second and rose in the third trimester [24,25,26,27]. Similarly, past studies showed that the levels of state anxiety reached their highest point in the first and third trimesters [28,29], whereas other studies reported that the scores rose significantly in the third trimester compared to the first and second trimesters [30,31].

### 1.2. The Role of Temperaments

It is noteworthy that the progress of psychological health during the perinatal period seems to be influenced by specific temperaments [32], considered the temporally stable dimension of personality [33]. Affective temperaments have been conceptualized by Akiskal and colleagues [34], who delineated hyperthymic, cyclothymic, irritable, depressive, and anxious temperaments [35,36]. Previous studies have already demonstrated the significant role of stable psychological traits, such as personality traits, on women’s mental health after delivery. For instance, specific traits, such as neuroticism, could not only be differentially associated with the severity of postpartum depression but could also act as mediators in the relationships between depression and other psychopathological dimensions, such as bipolar features [37]. Although the literature has noted the associations between temperaments and mood disorders [33,38], less research has focused on the association between temperaments and mood disorders during the perinatal period [39,40,41].

### 1.3. Research Gaps and Aims

Studies examining the associations between perinatal depression, anxiety, and specific temperaments during pregnancy are currently lacking in the literature. Within these associations, the different forms of anxiety should be considered simultaneously to identify their specific role. Therefore, this study aims to investigate the associations between the severity of perinatal depression, generalized anxiety disorder (GAD), state and trait anxiety, and temperament in women during the first, second, and third trimesters of pregnancy using a network analysis approach. Moreover, this study evaluated the differences in the severity of perinatal depression and anxiety dimensions (state, trait, and GAD) between women in the first, second, and third trimesters of pregnancy.

The relevance of perinatal mental health is also highlighted, considering its long-term effects. Indeed, when left untreated, perinatal mood is associated with adverse effects for women and their children, such as poor adherence to medical care, poor nutrition, loss of interpersonal resources, smoking, and substance abuse [42]. Depression and anxiety during pregnancy were found to be risk factors for children’s neurological, cognitive, and emotional development [1,43,44,45,46,47]. In addition, it is necessary to consider that the mother’s mental disorders, often occurring with the paternal ones [48], might persist after birth, negatively affecting the quality of interactions between the parent and infant [49].

## 2. Materials and Methods

This study used a cross-sectional design and was conducted at the Obstetrics and Gynecology Unit of Policlinico Universitario A. Gemelli between January 2022 and July 2023. A convenience sampling method was used to recruit the participants, consisting of pregnant women who presented themselves at the Unit and voluntarily decided to participate. The inclusion criteria were as follows: (1) Italian nationality, (2) current pregnancy, and (3) age 18 years or older. The exclusion criterion was (1) being on psychopharmacological treatment. Ethical approval was obtained from the Ethics Committee of the Fondazione Policlinico Universitario Agostino Gemelli IRCCS, Università Cattolica del Sacro Cuore of Rome, Italy (protocol ID: 2221). Written informed consent was obtained from all participants. Clinical assessment was performed during the first visit, and the following psychometric scales were administered: the Edinburgh Postnatal Depression Scale (EPDS); the seven-item Generalized Anxiety Disorder (GAD-7); the State-Trait Anxiety Inventory 1 and 2 (STAI-1 and STAI-2); and the Temperament Evaluation of Memphis, Pisa, Paris, and San Diego (TEMPS-A). When psychiatric evaluations revealed the presence of depression, the participants were offered further diagnostics and treatment. Depending on the evidence found, they were offered psychotherapeutic and, when necessary, psychopharmacological treatment.

### 2.1. Measures

The following psychometric scales were used:

Edinburgh Postnatal Depression Scale (EPDS): A 10-item self-assessment questionnaire that measures the risk of depression during the peripartum period in the last week. Higher scores indicate a higher risk of perinatal depression. The Italian validation of the EPDS was used [50]. Initially developed for the identification of postpartum depression [51], the EPDS was later validated for prenatal screening as well [52,53]. The questionnaire refers to how the woman felt in the last 7 days, and each item is scored on a Likert-scale from 0 to 3, with each point receiving variable labels. Items 1 and 2 assess anhedonia, 3 guilt, 4 anxiety, 5 fear or panic, 6 helplessness, 7 sleep disorders, 8 sadness, 9 tendency to cry, and 10 tendency towards self-harm. Items 1, 2, and 4 are scored according to an increasing severity gradient, while all others are scored reversely. Higher scores indicate a higher risk of perinatal depression.

Generalized Anxiety Disorder-7 (GAD-7): A 7-item self-report questionnaire to assess symptoms of generalized anxiety disorder. Higher scores indicate a higher level of anxiety. The GAD [54] consists of seven items that assess generalized anxiety disorder symptoms (“Feeling nervous, anxious, or on edge?”, “Not being able to stop or control worrying?”, “Worrying too much about different things?”, “Trouble relaxing?”, “Being so restless that it is hard to sit still?”, “Becoming easily annoyed or irritable?”, and “Feeling afraid as if something awful might happen?”), with four response options each, where each item is scored from 0 to 3, with 21 being the maximum score and the presence of mild symptoms detected from 5 points.

State-Trait Anxiety Inventory (STAI): This inventory measures state anxiety (S-anxiety) and trait anxiety (T-anxiety) separately. Higher scores indicate a higher level of stress. Charles Spielberger developed his concept of state and trait anxiety [55] after Cattell and Schneier [56] to measure two constructs, one responsive to environmental change (state) and the other relatively invariant (trait) [57]. S-Anxiety is a transitory response to an event perceived as adverse, characterized by feelings of tension, apprehension, nervousness, and worry. T-Anxiety is a more stable predisposition to perceive stressful situations as dangerous or threatening [55]. The inventory consists of two self-rated sheets with 20 statements each. Every item is scored from 1 to 4 according to Likert scales, with the state referring to “how you feel right now”, with responses being 1 = Not at all, 2 = Somewhat, 3 = Moderately so, and 4 = Very much so, and the trait referring to “how you generally feel”, with responses being 1 = Almost never, 2 = Sometimes, 3 = Often, and 4 = Almost always. Higher scores indicate higher anxiety levels. The validated Italian version of the STAI-Y was administered [58].

Temperament Evaluation of Memphis, Pisa, Paris, and San Diego—Auto-questionnaire (TEMPS-A): A self-assessment questionnaire of five affective temperaments: depressive, cyclothymic, hyperthymic, irritable, and anxious. Higher scores indicate a predominant temperament. The TEMPS-A contains 110 “true” or “false” items. The highest score is considered to indicate the prevailing temperament [34]. Dimensions of affective temperament are associated with specific behavioral features, e.g., a depressive temperament is connected with a tendency to pessimism, worry, guilt-proneness, difficulties in experiencing joy, and preoccupation with inadequacy, failure, and negative events. A cyclothymic temperament is characteristic of persons presenting unstable moods, labile self-esteem, and overconfidence, alternating with low self-confidence. A hyperthymic temperament characterizes people with high openness for novel experiences, high activity, and leadership, as well as higher risk behaviors, stress resistance, and grandiosity. The irritable dimension may manifest itself in dysphoric, explosive reactions and restlessness. The anxious temperament is associated with a higher risk of anxiety disorders and defense reactions [34]. We used the validated Italian version of TEMPS-A [59].

### 2.2. Statistical Methods

#### 2.2.1. Descriptive Analyses

Initially, descriptive analyses of the three groups were performed. Possible differences in sociodemographic and anamnestic data were assessed through analysis of variance (ANOVA) (age) and chi-square tests (occupation and level of education, being primiparas, previous abortion, and having had a history of psychiatric symptomatology).

#### 2.2.2. Correlation Analyses

Correlation analyses (Pearson’s r) were conducted for each group to evaluate the correlations between EPDS, generalized anxiety disorder, state and trait anxiety (assessed with GAD-7, STAI-1, and STAI-2, respectively), and temperaments. The sociodemographic and anamnestic factors found to be non-homogeneously distributed between groups were inserted as control variables in the correlation analyses.

#### 2.2.3. Network Analyses

A network analysis with EPDS, anxiety (GAD-7, STAI-1, and STAI-2), and women’s temperaments in the first, second, and third trimesters of pregnancy was conducted to visually explore the simultaneous associations between the variables [60]. A network is constituted by “nodes” that represent the variables. The nodes are connected by “edges”, representing statistical relationships, which can differ in the strength of the connection (edge weight). Moreover, the blue edges show a positive relationship, while the red edges are negative. Thick and denser colored edges show a strong relationship, whereas thin and less dense edges are weak [61]. The Gaussian graphical model estimation method, the extended Bayesian information criterion (EBIC), the graphical least absolute shrinkage, and selection operator (GLASSO) algorithms were applied [62]. Importantly, using EBIC with GLASSO improves the ratio between the number of correctly identified edges and the total number of edges for sparse graphs [61]. A tuning hyperparameter of 0.25 was set to increase the network’s sensitivity. A minimum value of 0.03 was considered to interpret the results [63]. JASP software (2021) was used for the statistical analyses.

In the present study, the centrality indices were evaluated to assess the relevance of a node in the network [64], considering the indices of closeness, strength, and expected influence. The closeness index quantifies the node’s indirect relationships with all the other nodes within the network [65]. A strength index indicates how a node is directly associated with other nodes [61]. The expected influence index for a node estimates a node’s influence on its neighbors [66]. The edge weight accuracy was assessed using non-parametric bootstrapping (1000 replicates) to compute 95% confidence intervals (CIs). The stability of the centrality indices was examined using case-dropping subset bootstrapping (1000 replicates). The centrality stability (CS) coefficient was assessed and considered acceptable when it was at least 0.25 [61]. RStudio was used to evaluate CS [67].

#### 2.2.4. Analyses of Variance and Covariance

ANOVAs were conducted on EPDS, GAD-7, STAI-1, and STAI-2 by including trimesters (first vs. second vs. third) as a between-subject factor. Lastly, analyses of covariance (ANCOVAs) were conducted to evaluate how the sociodemographic and anamnestic data found to be significantly different between the three groups affected the results of the ANOVAs. Levene’s test checked the assumption of the equality of variances, and a Bonferroni correction was applied to post hoc comparisons.

## 3. Results

Two hundred and eighty participants signed the informed consent form and participated in the study. At the time of the study, 11 women were receiving psychotropic medications and, considering the possible influence on the test results, were excluded from further analyses, leading to a total sample of 269 participants. Among them, 31 (M_age = 32.9; SD_age = 5.3; range 21–42) were in the first trimester of pregnancy, 184 (M_age = 35.3; SD_age = 5.4; range 19–45) were in the second, and 54 (M_age = 34.4; SD_age = 5.6; range 25–51) were in the third. Regarding occupational status, there were 22 (71.0%) workers and 9 (29.0%) unemployed women in the first trimester, 140 (76.1%) workers and 44 (23.9%) unemployed in the second, and 42 (77.8%) workers and 12 (22.2%) unemployed in the third trimester. In addition, among women in the first trimester of pregnancy, 16 (51.6%) had a degree, 12 (38.7%) had a high school degree, and 3 (9.7%) had a middle school degree. In total, 92 women (50.0%) in the second trimester of pregnancy had a degree, 81 (44.0%) had a high school degree, and 11 (6.0%) had a middle school degree; among women in the third trimester of pregnancy, 33 (61.1%) had a degree, 18 (33.3%) had a high school degree, and 3 (5.6%) had a middle school degree. A total of 17 (54.8%) women in the first, 73 (39.7%) in the second, and 21 (38.9%) in the third trimester of pregnancy were primiparas, whereas 8 (25.8%) women in the first, 53 (28.8%) women in the second, and 19 (35.2%) women in the third trimester of pregnancy, had a previous miscarriage. Lastly, 11 (35.5%) women in the first, 25 (13.6%) women in the second, and 5 (9.3%) women in the third trimester of pregnancy had a history of psychiatric symptomatology.

Concerning the differences in socio-demographic factors between the three groups, the ANOVA performed on age by including trimesters (first vs. second vs. third) as a between-subject factor was not significant (F(2,266) = 2.671, partial eta = 0.020, *p* > 0.05). In addition, the chi-square tests showed no differences between the three groups on occupational (χ^2^ (2, N = 269) = 0.5, *p* > 0.05) and educational (χ^2^ (4, N = 269) = 2.8, *p* > 0.05) status, being primiparas (χ^2^ (2, N = 269) = 2.6, *p* > 0.05), or having had a previous miscarriage (χ^2^ (2, N = 269) = 1.1, *p* > 0.05). On the contrary, the proportion of women who had a history of psychiatric symptomatology differed by groups (χ^2^ (2, N = 269) = 11.7, *p* = 0.003).

Table 1 shows the partial correlations between EPDS, STAI-1, STAI-2, GAD-7 and the depressive, cyclothymic, hyperthymic, irritable, and anxious temperaments among women in the first trimester of pregnancy, with the history of psychiatric symptomatology as a control variable (Table 1, “A”). EPDS scores were positively correlated with both STAI-1 and STAI-2, GAD-7, and depressive, irritable, and anxious temperaments. STAI-1 was positively correlated with STAI-2, GAD-7, and depressive, irritable, and anxious temperaments. Similarly, STAI-2 was positively correlated with GAD-7 and depressive, irritable, and anxious temperaments. GAD-7 scores were positively correlated with depressive, irritable, and anxious temperaments. Finally, depressive temperament was positively correlated with irritable and anxious temperaments, cyclothymic temperament was positively correlated with anxious temperament, and irritable temperament was positively correlated with anxious temperament.

For women in the second trimester of pregnancy (Table 1, “B”), EPDS scores were positively correlated with STAI-1 and STAI-2, GAD-7, and all temperaments except the hyperthymic. STAI-1 was positively correlated with STAI-2, GAD-7, and all the temperaments. STAI-2 was positively correlated with GAD-7 and with all the temperaments. GAD-7 scores positively correlated with all the temperaments. Lastly, depressive temperament was positively correlated with cyclothymic, irritable, and anxious temperaments, cyclothymic temperament was positively correlated with irritable and anxious temperaments, and irritable temperament was positively correlated with anxious temperament.

Lastly, regarding women in the third trimester of pregnancy (Table 1, “C”), EPDS was positively correlated with STAI-1, STAI-2, GAD-7, and cyclothymic, irritable, and anxious temperaments. STAI-1 was positively correlated with STAI-2, GAD-7, and cyclothymic and anxious temperaments. STAI-2 was positively correlated with GAD-7 and all temperaments except for hyperthymic. GAD-7 was positively correlated with all the temperaments except for hyperthymic. Lastly, depressive temperament was positively correlated with cyclothymic, irritable, and anxious temperaments, cyclothymic temperament was positively correlated with irritable and anxious temperaments, and irritable temperament was positively correlated with anxious temperament.

These results suggest that higher depressive symptoms are associated with increased anxiety and certain temperamental traits.

**Figure 1:**
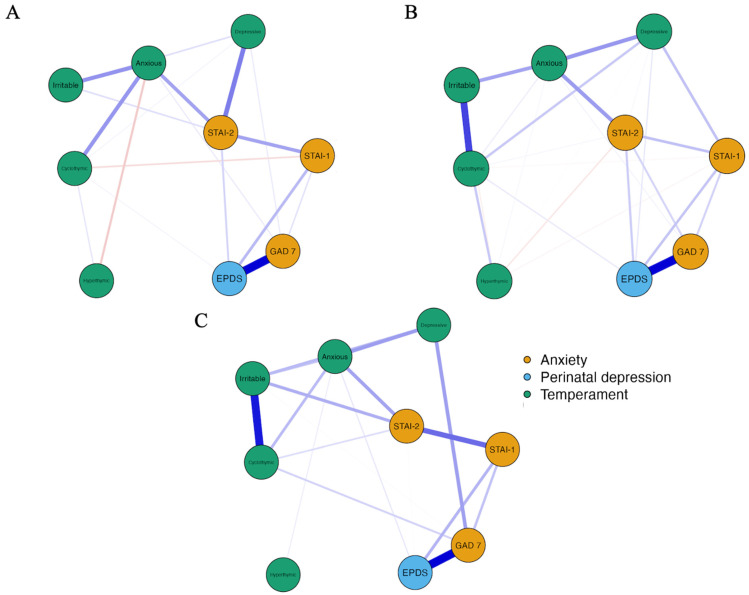
Network structures (partial correlations) of the associations between Perinatal depression (in blue), anxiety (in ochre), and temperaments (in green) in women in the first (**A**), second (**B**), and third (**C**) trimesters of pregnancy. Note. Blue edges represent positive associations, and red edges represent negative associations. Thicker and denser colored edges represent stronger associations, and thinner and less dense colored edges represent weaker associations. “EPDS” = Edinburgh Postnatal Depression Scale; “STAI” = State-Trait Anxiety Inventory; “GAD 7” = Generalized Anxiety Disorder.

Figure 1 shows the network models depicting the partial correlations among EPDS, anxiety measures (STAI-1, STAI-2, and GAD-7), and temperaments (depressive, cyclothymic, hyperthymic, irritable, and anxious) for pregnant women in the first (A), second (B), and third (C) trimesters. Each model consisted of 9 nodes. In model A (first trimester), there were 21 out of 36 non-zero edges with a sparsity index of 0.417, indicating moderate connectivity. Model B (second trimester) showed higher connectivity with 26 non-null edges and a sparsity index of 0.278. Model C (third trimester) had similar connectivity to Model A, with 20 non-null edges and a sparsity index of 0.444. These results suggest that the associations between psychological variables and temperaments vary across trimesters.

The weight matrices of associations between the variables in the network models are shown in the Supplementary Materials (Table S1). In Model A, related to the women in the first trimester of pregnancy, the EPDS showed the strongest positive association with GAD-7, followed by STAI-1 and STAI-2 and cyclothymic temperament. Moreover, GAD-7 was positively correlated with STAI-1 and depressive and anxious temperaments. STAI-1 was negatively correlated with cyclothymic temperament and positively with STAI-2. Lastly, STAI-2 was positively correlated with depressive and anxious temperaments.

In Model B, related to the women in the second trimester of pregnancy, the EPDS showed positive associations with GAD-7, STAI-1, STAI-2, and cyclothymic temperament. In addition, in Model B, the EPDS was positively associated with depressive temperament. GAD-7 was positively correlated with STAI-1 and STAI-2 and anxious temperament. STAI-1 was positively correlated with STAI-2 and depressive temperament and negatively with hyperthymic and cyclothymic temperaments. STAI-2 showed positive associations with depressive, cyclothymic, and anxious temperaments and negative associations with hyperthymic temperament.

In the last Model, C, related to the women in the third trimester of pregnancy, the EPDS showed a positive association with GAD-7, STAI-1, STAI-2, and anxious temperament. GAD-7 showed positive associations with STAI-1 and depressive temperament. STAI-1 was positively associated with STAI-2. STAI-2 was positively associated with cyclothymic and anxious temperaments.

**Figure 2:**
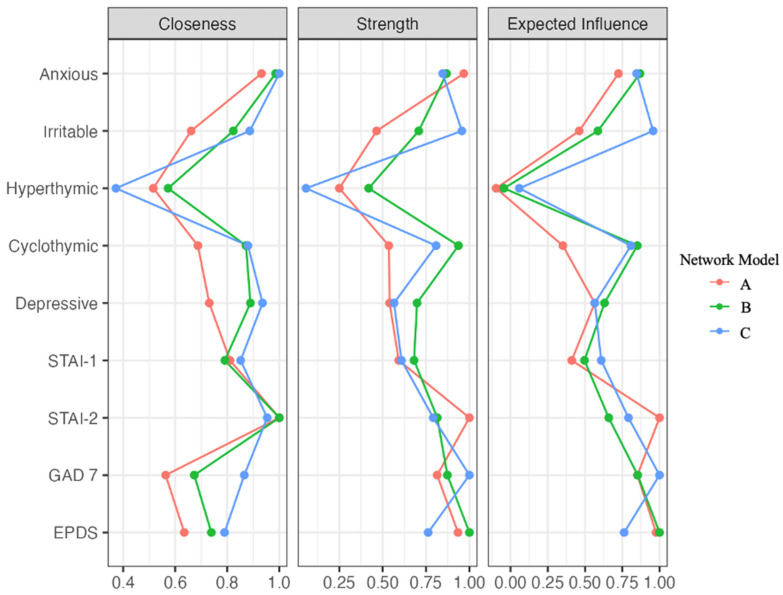
Centrality plots for the associations among variables among the network models related to women in the first (**A**), second (**B**), and third (**C**) trimester of pregnancy. Note. “STAI” = State-Trait Anxiety Inventory; “GAD 7” = Generalized Anxiety Disorder Questionnaire 7; “EPDS” = Edinburgh Postnatal Depression Scale.

Figure 2 shows the diagrams of the centrality indices. In Model A (first trimester), STAI-2 had the highest centrality index for closeness (z = 1), followed by anxious temperament (z = 0.93), STAI-1 (z = 0.81), and depressive temperament (z = 0.73). In Model B (second trimester), STAI-2 again showed the highest closeness index (z = 1), followed by anxious temperament (z = 0.99), depressive (z = 0.89), and cyclothymic (z = 0.87) temperaments. In Model C (third trimester), anxious temperament had the highest closeness index (z = 1), followed by STAI-2 (z = 0.95), depressive (z = 0.94), and cyclothymic temperament (z = 0.88). These results suggest that the STAI-2 and anxious temperament are central to understanding the network of psychological variables during pregnancy.

In Model A (first trimester), STAI-2 had the highest Strength index (z = 1), followed by anxious temperament (z = 0.97), EPDS (z = 0.93), and GAD-7 (z = 0.81). In Model B (second trimester), EPDS had the highest Strength value (z = 1), followed by cyclothymic temperament (z = 0.94), GAD-7 (z = 0.8791), and anxious temperament (z = 0.8790). Finally, in Model C (third trimester), the GAD-7 (z = 1) and the irritable (z = 0.96), anxious (z = 0.85), and cyclothymic (z = 0.81) temperaments showed the highest Strength values. The findings highlight that, in particular, STAI-2, EPDS, and GAD-7 could be strongly and directly associated with other variables during pregnancy.

The STAI-2 (z = 1), the EPDS (z = 0.98), the GAD-7 (z = 0.85), and the anxious temperament (z = 0.72) had the highest Expected Influence values in Model A (first trimester). In Model B (second trimester), the EPDS (z = 1), the anxious temperament (z = 0.87), the GAD-7 (z = 0.85), and the cyclothymic (z = 0.85) temperament had the highest values. Finally, in Model C (third trimester), the GAD-7 (z = 1) and the irritable (z = 0.96), anxious (z = 0.85), and cyclothymic (z = 0.81) temperaments showed the highest Expected Influence values. The results suggest an important role of EPDS and GAD-7 in influencing the variables close to them in the networks.

Figures S1 and S2 show the accuracy of the edges’ weights and the stability of centrality indices (CS), respectively (Supplementary Materials). In Model A, the CS of closeness was 0.19, the expected influence was 0.45, and the strength was 0.24 (Figure S2A). In Model B, the CS of closeness was 0.28, the expected influence was 0.75, and the strength was 0.59 (Figure S2B). In Model C, closeness showed a CS of 0, an expected influence of 0.52, and strength of 0.37 (Figure S2C). Considering the threshold of 0.25 [61], these findings suggested that expected influence values were stable across the three models, whereas strength was interpreted in Model B and C and closeness only in Model B.

Concerning the differences in EPDS between the three trimesters, ANOVA results did not show a significant main effect of trimester on EPDS scores (F(2,266) = 2.820, partial eta η^2^ = 0.021, *p* = 0.06). The ANOVA performed on GAD-7 by including trimesters as a between-subject factor showed a significant main effect of trimester on GAD-7 scores (F(2,266) = 3.202, partial eta η^2^ = 0.024, *p* = 0.04). Post hoc comparisons revealed that women in the first trimester (M = 6.68, SD = 3.32) had significantly higher GAD-7 scores than women in the second (M = 5.04, SD = 3.29) trimester (*p* = 0.03). The ANCOVA results showed that the main effect of trimester was not significant on the GAD-7 scores when controlling for the history of psychiatric symptomatology (F(2,266) = 1.616, partial eta η^2^ = 0.012, *p* > 0.05). Lastly, no differences were found in trait and state anxiety between women in the three trimesters.

## 4. Discussion

This study examined the associations between perinatal depression (EPDS), generalized anxiety disorder (GAD), state and trait anxiety, and temperament across different trimesters of pregnancy. Network analysis revealed that EPDS and GAD were strongly associated across all trimesters, emphasizing their central role in perinatal mental health. Moreover, the study evaluated the differences in the severity of perinatal depression and anxiety between women in the three trimesters of pregnancy, revealing a higher severity of GAD in women in the first trimester of pregnancy than women in the second trimester. However, when controlling for the history of psychiatric symptoms, this difference was no longer significant, confirming the importance of considering the psychiatric history of pregnant women during pregnancy screening.

The network analysis showed that EPDS and GAD were the nodes most strongly connected across the three different groups. These results confirm previous studies indicating a high comorbidity between depression and anxiety disorders during pregnancy [68]. The high scores for the expected impact of EPDS and GAD suggest that they have a significant impact on mental health during pregnancy. This is consistent with studies reporting high levels of dissatisfaction and unfulfilled expectations in pregnant women with these disorders [69,70]. Additionally, EPDS showed the strongest expected influence in the first and second trimester networks, while GAD dominated in the third-trimester network. This suggests that depressive symptoms are more influential early and mid-pregnancy, while anxiety symptoms become more important later in pregnancy. These patterns emphasize the importance of monitoring and treating both depressive and anxiety symptoms during pregnancy. These symptoms were theorized as a relevant component of the wider experience of loss experienced by some women, developed in the context of the many daily, physical, and role changes inherent to pregnancy [71]. The present study additionally found that women in the first trimester showed the highest severity of GAD, significantly higher than women in the second trimester of pregnancy, confirming previous research [29]. In this regard, the second trimester has been proposed to be a time of moderately greater stability than the initial adjustment to the new state experienced in the first trimester [72].

In all three networks, the EPDS was positively associated with both state and trait anxiety, suggesting that higher levels of depressive symptoms are closely associated with immediate and chronic anxiety responses. These associations were particularly strong in the first trimester, suggesting that early pregnancy may be a critical period for the co-occurrence of depression and anxiety. Coherently, some authors have argued that the anxiety dimensions could act as risk factors for the severity of perinatal depression [73,74,75]. The present study also showed that the strength of these associations was greater in the network related to women in the first trimester. In addition, trait anxiety in women in the first trimester shared with EPDS the higher expected influence value, confirming its relevance. These findings supported the importance of assessing anxiety symptoms during pregnancy as, when compared to depression, anxiety disorders are generally less recognized during the perinatal period [76].

Interestingly, it should be noted that state and trait anxiety dimensions showed stronger associations with EPDS than with GAD in all the networks. Although longitudinal studies are needed to verify the evolution of the magnitude of the associations and the direction of the influence between these dimensions during pregnancy, the results would suggest that the state and trait anxiety dimensions might have greater relevance within the depressive symptomatology during pregnancy.

The EPDS was positively associated with a cyclothymic temperament, suggesting that individuals with this temperament are more prone to depressive symptoms during pregnancy. Cyclothymic temperament, which is characterized by mood instability, could exacerbate emotional challenges during pregnancy. In addition, anxious and depressive temperaments were frequently associated with both EPDS and GAD, suggesting that they play an important role in perinatal mental health. Moreover, cyclothymic temperament had a high strength index, indicating that it was particularly directly connected to the other nodes. In this regard, cyclothymic temperament was found to be the most representative temperament in patients with major depressive disorder [77]. Moreover, it has been seen to be a risk factor for postpartum depression [39]. Considering the associations found in the literature between cyclothymic temperament and suicidal ideations or attempts [78,79], the findings of the present study suggested that relevant clinical attention should be given to these temperamental characteristics. In the second network, in addition to cyclothymic, EPDS was also associated with depressive temperament, whereas in the third network, EPDS was only associated with anxious temperament. Previous research found associations between depressive and anxious temperaments and major depression diagnosis [80,81], and the results of the present study would suggest that such temperament types might play a specific role in women’s depressive symptoms and that this could be associated with the specific trimester of pregnancy.

The present study sheds light also on the associations between GAD and temperaments. Specifically, in the first network, GAD was positively associated with anxious and depressive temperament, and in the second network, GAD was positively associated only with the anxious temperament. In this regard, the anxious temperament was shown to be a predictor of disorders within the anxiety clusters [39,82]. In this regard, past research hypothesized that GAD could be conceptualized as a temperamental style underlying chronic and generalized anxiety [83,84,85]. Interestingly, in the third network, GAD was positively associated with depressive, cyclothymic, and irritable temperaments. This would suggest that toward the end of pregnancy, temperamental depressive elements might be a greater risk factor than others for anxiety symptomatology, although longitudinal research designs are needed to test this hypothesis.

Overall, cyclothymic, depressive, and anxious were the temperaments most frequently associated with EPDS and GAD. Interestingly, trait anxiety was visually located at the center of the three networks. Coherently, this would indicate that this dimension could play a central role across the three trimesters of pregnancy. Oppositely, hyperthymic temperament was in the periphery of the network across the three networks, reporting low centrality indices, and, in the second network, was negatively associated with state and trait anxiety. Interestingly, the hyperthymic temperament score was found to be negatively correlated with postpartum depression, suggesting that hyperthymic temperament is a protective factor for the post-birth period [38,86,87]. Moreover, the hyperthymic temperament appears to have a protective effect on several mental disorders [39]. Considering the findings of the present study, it could be hypothesized that this temperament could potentially act as a protective factor of mental health during pregnancy.

Despite the merits of the present study, several limitations must be considered. The main limitation of this study is its cross-sectional design, which does not allow causal conclusions to be drawn. In this regard, inferences about the directionality of relationships between variables cannot be drawn. In addition, recent studies criticized the validity of centrality hypotheses in studies with a cross-sectional design [88,89,90]. Consequently, the results concerning the centrality indices should be considered cautiously. An important limitation is the sampling strategy, which may have led to a selection bias. In particular, the research’s results conducted on convenience samples can only be generalized to the conveniently accessible population from which the sample was drawn. Consequently, extreme caution is required in generalizing the results of the present study to all pregnant women. In addition, this selection method could be associated with the differences in the size of women’s groups between the three trimesters, where fewer patients were recruited in the first and third trimesters than in the second trimester. This is probably related to the period of pregnancy. Indeed, patients during the second trimester went to a third-level institution such as Policlinico A. Gemelli to carry out in-depth examinations related to pregnancy and the fetus. In particular, more screenings are planned during this trimester to investigate fetal growth and possible malformations. It should also be noted that the present study included self-report measures, possibly affecting the responses due to social desirability and common method biases. Procedural controls were implemented to reduce such potential sources of bias, providing participants with clear instructions and ensuring their anonymity [91]. In addition, multiple other variables not assessed in the present study could influence depression and anxiety [92]. Future studies should use longitudinal design and predictive statistical modeling to understand the temporal dynamics and causal relationships between perinatal depression, anxiety, and temperamental traits. Investigating additional factors such as perceived stress, social support, and adverse childhood experiences could provide a more comprehensive understanding of the influences on perinatal mental health. The inclusion of objective measures and larger, more balanced sample sizes across all trimesters would also improve the robustness of future research.

## 5. Conclusions

In conclusion, the present study revealed the interrelationships between the severity of depressive symptoms, anxiety, and temperaments in women in the first, second, and third trimesters of pregnancy. In all three groups, perinatal depression and GAD showed the strongest associations, underscoring the strong comorbidity between the two conditions, and showed a high relevance in the networks. Both state and trait anxiety also had positive associations with depression severity and GAD, suggesting their role as possible risk factors. Relevantly, temperaments were differently associated within the network between the three groups. These results would suggest interesting clinical implications during pregnancy, identifying direct and indirect relationships between the investigated variables. This is also important considering the higher levels of severity of GAD found in first-trimester women.

## Figures and Tables

**Table 1:** Partial correlations (Pearson’s r) between EPDS, STAI-1, 2, GAD-7, and depressive, cyclothymic, hyperthymic, irritable, and anxious temperaments related to women in the first (A), second (B), and third (C) trimester of pregnancy, after controlling for the history of psychiatric symptomatology.

(A) Variable	1	2	3	4	5	6	7	8
1. EPDS	—							
2. STAI-1	0.803 ***	—						
3. STAI-2	0.776 ***	0.767 ***	—					
4. GAD-7	0.956 ***	0.774 ***	0.749 ***	—				
5. Depressive	0.612 ***	0.513 **	0.762 ***	0.639 ***	—			
6. Cyclothymic	0.323	0.150	0.241	0.316	0.308	—		
7. Hyperthymic	−0.116	−0.176	−0.229	−0.164	−0.115	0.107	—	
8. Irritable	0.402 *	0.406 *	0.603 ***	0.457 *	0.522 **	0.189	−0.171	—
9. Anxious	0.602 ***	0.561 **	0.747 ***	0.645 ***	0.594 ***	0.454 *	−0.262	0.632 ***
**(B) Variable**	**1**	**2**	**3**	**4**	**5**	**6**	**7**	**8**
1. EPDS	—							
2. STAI-1	0.680 ***	—						
3. STAI-2	0.708 ***	0.626 ***	—					
4. GAD-7	0.885 ***	0.666 ***	0.696 ***	—				
5. Depressive	0.556 ***	0.529 ***	0.555 ***	0.500 ***	—			
6. Cyclothymic	0.465 ***	0.293 ***	0.459 ***	0.408 ***	0.507 ***	—		
7. Hyperthymic	−0.123	−0.181 *	−0.174 *	−0.155 *	−0.126	0.096	—	
8. Irritable	0.366 ***	0.273 ***	0.426 ***	0.343 ***	0.425 ***	0.684 ***	−0.054	—
9. Anxious	0.556 ***	0.469 ***	0.642 ***	0.555 ***	0.623 ***	0.553 ***	−0.022	0.579 ***
**(C) Variable**	**1**	**2**	**3**	**4**	**5**	**6**	**7**	**8**
1. EPDS								
2. STAI-1	0.537 ***							
3. STAI-2	0.439 ***	0.567 ***						
4. GAD-7	0.778 ***	0.531 ***	0.445 ***					
5. Depressive	0.267	0.257	0.366 **	0.513 ***				
6. Cyclothymic	0.383 **	0.306 *	0.593 ***	0.546 ***	0.402 **			
7. Hyperthymic	−0.082	−0.100	−0.066	−0.035	0.003	0.027		
8. Irritable	0.320 *	0.240	0.599 ***	0.488 ***	0.513 ***	0.811 ***	0.027	
9. Anxious	0.500 ***	0.451 ***	0.632 ***	0.546 ***	0.587 ***	0.666 ***	0.109	0.684 ***

Note. * *p* < 0.05, ** *p* < 0.01, *** *p* < 0.001. “EPDS” = Edinburgh Postnatal Depression Scale; “STAI” = State-Trait Anxiety Inventory; “GAD-7” = Generalized Anxiety Disorder Questionnaire.

## Data Availability

The data presented in this study are available on request from the corresponding author upon reasonable request.

## Supplementary Materials

The following supporting information can be downloaded at: https://www.mdpi.com/article/10.3390/jcm13133957/s1, Table S1: Weights matrix of associations (partial correlations) between EPDS, anxiety (GAD7, STAI-1 and STAI-2), and temperaments between the networks related to women in the first trimester of pregnancy (A), women in the second trimester of pregnancy (B), and women in the third trimester of pregnancy (C); Figure S1: The bootstrapped confidence intervals of the network models of women in the first (A), second (B), and third (C) trimesters of pregnancy. The x-axis represents the edges, and every line on the y-axis represents a specific edge. The red line shows the estimate of each edge weight. The 95% Confidence Intervals of edge weights are represented in a gray area; Figure S2: Stability of centrality indices related to the network model of women in the first trimester of pregnancy (A), women in the second trimester of pregnancy (B), and women in the third trimester of pregnancy (C). The percentage of cases of the original sample is represented in the x-axis. The average correlations between the centrality indices from the original network and those from the networks replicated after dropping cases are shown in the y-axis. The red lines indicate the correlations of closeness, green lines indicate expected influence, and blue lines the strength, while areas indicate 95% Confidence Intervals (CIs).
