# Supplementary material for: A Network Analysis of Perinatal Depression, Anxiety, and Temperaments in Women in the First, Second, and Third Trimesters of Pregnancy
Source: J Clin Med. 2024 Jul 5;13(13):3957. doi: 10.3390/jcm13133957 (PMC11242710; doi:10.3390/jcm13133957)

Table S1. Weights matrix of associations (partial correlations) between EPDS, anxiety (GAD7, STAI 1 and 2), and temperaments, between the networks related to women in the first trimester of pregnancy (A), women in the second trimester of pregnancy (B), and women in the third trimester of pregnancy (C).

	A									B									C								
**Variable**	**1**	**2**	**3**	**4**	**5**	**6**	**7**	**8**	**9**	**1**	**2**	**3**	**4**	**5**	**6**	**7**	**8**	**9**	**1**	**2**	**3**	**4**	**5**	**6**	**7**	**8**	**9**
1. EPDS	0.00	0.23	0.15	0.75	0.00	0.06	0.00	0.00	0.00	0.00	0.18	0.16	0.70	0.09	0.08	0.00	0.00	0.00	0.00	0.19	0.04	0.57	0.00	0.00	0.00	0.00	0.08
2. STAI-1	0.23	0.00	0.29	0.11	0.00	-0.13	0.00	0.00	0.00	0.18	0.00	0.19	0.14	0.19	-0.05	-0.06	0.00	0.00	0.19	0.00	0.34	0.16	0.00	0.00	0.00	0.00	0.01
3. STAI-2	0.15	0.29	0.00	0.00	0.40	0.00	-0.03	0.12	0.29	0.16	0.19	0.00	0.14	0.05	0.06	-0.09	0.00	0.28	0.04	0.34	0.00	0.00	0.00	0.10	0.00	0.20	0.22
4. GAD 7	0.75	0.11	0.00	0.00	0.09	0.00	0.00	0.00	0.09	0.68	0.14	0.14	0.00	0.00	0.00	-0.01	0.00	0.06	0.57	0.16	0.00	0.00	0.23	0.12	0.00	0.04	0.02
5. Depr	0.00	0.00	0.40	0.09	0.00	0.06	0.00	0.12	0.02	0.09	0.19	0.05	0.00	0.00	0.17	-0.04	0.00	0.29	0.00	0.00	0.00	0.23	0.00	0.00	0.00	0.18	0.24
6. Cyclot	0.06	-0.13	0.00	0.00	0.05	0.00	0.10	0.00	0.33	0.09	-0.05	0.06	0.00	0.17	0.00	0.17	0.50	0.08	0.00	0.00	0.10	0.12	0.00	0.00	0.00	0.52	0.18
7. Hyper	0.00	0.00	-0.03	0.00	0.00	0.10	0.00	-0.02	-0.18	0.00	-0.06	-0.09	-0.01	-0.04	0.17	0.00	-0.07	0.05	0.00	0.00	0.00	0.00	0.00	0.00	0.00	0.00	0.07
8. Irrit	0.00	0.00	0.12	0.00	0.10	0.00	-0.02	0.00	0.33	0.00	0.00	0.00	0.00	0.00	0.50	-0.07	0.00	0.26	0.00	0.00	0.20	0.04	0.18	0.52	0.00	0.00	0.16
9. Anx	0.00	0.00	0.29	0.09	0.02	0.33	-0.18	0.33	0.00	0.00	0.00	0.28	0.06	0.29	0.08	0.05	0.26	0.00	0.08	0.01	0.22	0.02	0.24	0.18	0.07	0.16	0.00

Note. "EPDS" = Edinburgh Postnatal Depression Scale; "STAI" = State-Trait Anxiety Inventory; "GAD 7" = Generalized Anxiety Disorder Questionnaire 7; "Depr" = Depressive temperament; "Cyclot" = Cyclothymic temperament; "Hyper" = Hypethymic temperament; "Irri" = Irritable temperament; "Anx" = Anxious temperament.

Figure S1. The bootstrapped confidence intervals of the network models of women in the first (A), second (B), and third (C) trimester of pregnancy. The x-axis represents the edges, and every line on the y-axis represents a specific edge. The red line shows the estimate of each edge weight. 95% Confidence Intervals of edge weights are represented in a gray area.

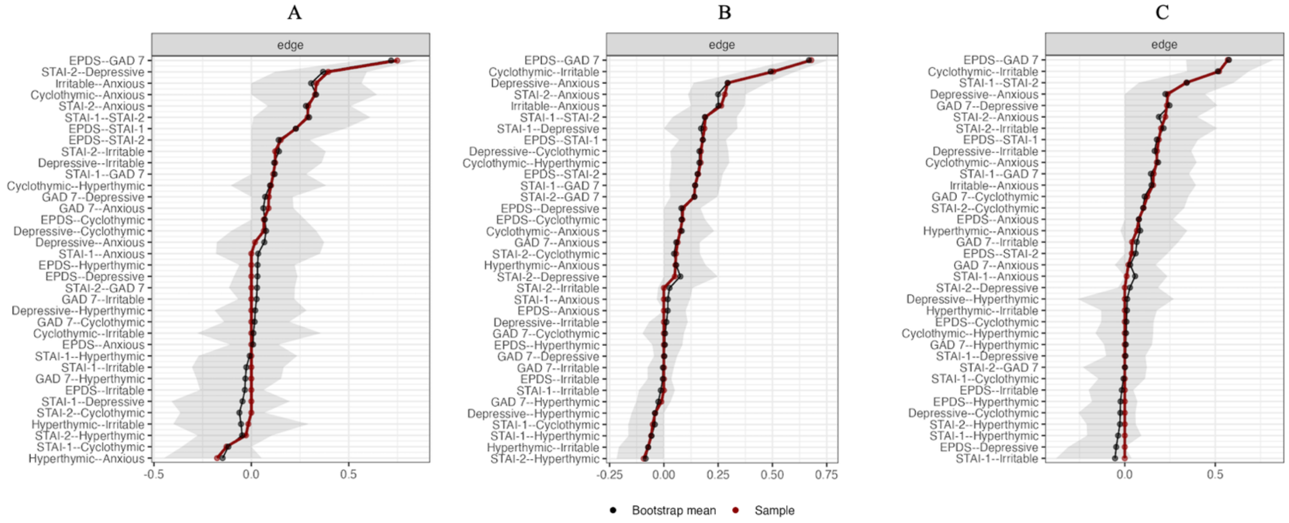

Note. “STAI” = State-Trait Anxiety Inventory; “GAD 7” = Generalized Anxiety Disorder Questionnaire 7; “EPDS” = Edinburgh Postnatal Depression Scale.

Figure S2. Stability of centrality indices related to the network model of women in the first trimester of pregnancy (A), women in the second trimester of pregnancy (B), and women in the third trimester of pregnancy (C). The percentage of cases of the original sample is represented in the x-axis. The average correlations between the centrality indices from the original network and those from the networks replicated after dropping cases are shown in the y-axis. The red lines indicate the correlations of closeness, green lines indicate expected influence, and blue lines the strength, while areas indicate 95% Confidence Intervals (CIs).

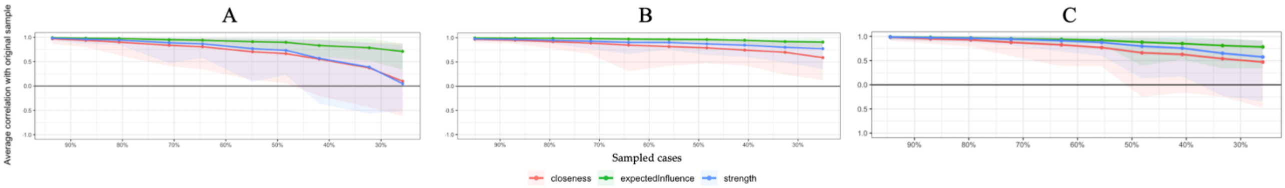

Supplement: Supplementary file 1 [file jcm-13-03957-s001.zip › jcm-3064530-supplementary.pdf]
